# ﻿The Grumpy dwarfgoby, a new species of *Sueviota* (Teleostei, Gobiidae) from the Red Sea

**DOI:** 10.3897/zookeys.1212.121135

**Published:** 2024-09-12

**Authors:** Viktor Nunes Peinemann, Lucía Pombo-Ayora, Luke Tornabene, Michael L. Berumen

**Affiliations:** 1 Red Sea Research Center, Division of Biological and Environmental Science and Engineering, King Abdullah University of Science and Technology, Thuwal 23955, Saudi Arabia King Abdullah University of Science and Technology Thuwal Saudi Arabia; 2 School of Aquatic and Fishery Sciences, and the Burke Museum of Natural History and Culture, University of Washington, 1122 NE Boat Street, Seattle, Washington, 98105, USA University of Washington Seattle United States of America

**Keywords:** Biodiversity, coral reef fish, Gobiidae, identification key, new species, Red Sea, *Sueviota*, taxonomy

## Abstract

A new gobiid species is described from ten specimens, 9.2 – 16.7 mm SL, collected from the Saudi Arabian Red Sea. The new species is most similar to *Sueviotapyrios* from the Gulf of Aqaba in the northern Red Sea. It differs from *S.pyrios* by having no large red spots on the dorsal and caudal fin elements, no elongate spines in the first dorsal fin, a shorter pelvic fin that does not reach the anus, branched pectoral fin rays, and a projecting lower jaw. The new species is further distinguished from all its congeners by a complete lack of cephalic sensory canals and pores. Specimens were found in small caves and overhangs at depths between 10 and 53 meters.

## ﻿Introduction

Winterbottom and Hoese (1988) described four species of tiny gobioid fishes that, despite their resemblance to the genus *Eviota* Jenkins, 1903, were placed in their own new genus *Sueviota* Winterbottom & Hoese, 1988. The main morphological differences between both genera are that most species of *Sueviota* have a basal pelvic membrane joining the fifth rays of the pelvic fins (vs pelvic fins well separate); the fifth pelvic rays themselves are very long (vs rudimentary or absent); and the fifth pelvic rays are branched (vs. unbranched). Since then, four additional species have been described and placed into *Sueviota*, with some of these species lacking one or more of the diagnostic characters that differentiate *Sueviota* from *Eviota*. There are currently eight valid species in *Sueviota* distributed throughout the Indo-Pacific, ranging from northwestern Australia, the South China Sea, Indonesia and Papua New Guinea to the Gulf of Aqaba in the northern Red Sea (Winterbottom and Hoese 1988; Allen et al. 2016; Allen and Erdmann 2017; Greenfield and Randall 2017; Greenfield et al. 2019).

During a diving expedition to explore the coral reef fish diversity on the Saudi Arabian coast of the central Red Sea near Al Lith (19.8375°N, 39.9296°E), we collected an unidentified dark red goby at a depth of 30 meters. The specimen was not observed during the dive but emerged anesthetized by the clove oil from a cave in a coral reef. Months later, in a different location on the Saudi Arabian coast of the central Red Sea close to Thuwal (22.4283°N, 38.9932°E), the same diver observed a similar-looking goby underneath a coral reef overhang covered with crustaceous coralline algae at a depth of 14 meters. Later that same year, the authors of this study were shown a goby belonging to the same species, collected at 53 meters from the central Red Sea at the latitude of Al Qunfudhah (18.9922°N, 40.6145°E). In August of the following year (2023), Darren Coker collected an orange-yellow goby from a rotenone station inside a cave at 10 meters at an inshore reef near Thuwal (22.2948°N, 39.0688°E). Finally, in June of 2024, seven additional specimens were collected at Maras Reef (19.8375°N, 39.9296°E) at depths of 20 and 30 meters. The COI fragments amplified from all specimens matched, and further morphological inspection confirmed the specimens belonged to the same undescribed *Sueviota* species.

## ﻿Material and methods

Counts, measurements, and morphological descriptions follow Lachner and Karnella (1980). Measurements were made to the nearest 0.1 mm using digital calipers or with AxioVision software using a ZEISS SteREO Discovery.V20 stereomicroscope with an AxioCam digital camera. To make the cephalic pores, fin rays, and scales easier to visualize, the specimens were dyed with Cyanine Blue 5R (acid blue 113) (Saruwatari et al. 1997). Holotype measurements and counts are provided in the description, followed by the range of the paratypes in parentheses. Type specimens were deposited at the ichthyology collections of the University of Washington (UW) and the California Academy of Sciences (CAS).

A micro-computed tomography scan (μCT) of UW 203365 was taken at the Karel F. Liem Bio-Imaging Center in Friday Harbor Laboratories, WA, USA, using a Bruker Skyscan 1173. The imaging parameters included a current of 123 uA, a voltage of 65 kV, and rotation steps of 0.25 degrees. The raw image stack was reconstructed using NRecon from Bruker and subsequent segmentation was conducted in 3D Slicer v.5.2.2 with the SlicerMorph extension (Kikinis et al. 2013; Rolfe et al. 2021).

Collections were done under the approved ethics protocol number 20IAUCUC05 issued by the Institutional Animal Care and Use Committee (IACUC) of the King Abdullah University of Science and Technology (KAUST).

## ﻿Results

### ﻿Taxonomic accounts

#### ﻿ Sueviota aethon

Taxon classificationAnimaliaPerciformesGobiidae

Nunes Peinemann, Pombo-Ayora & Tornabene sp. nov.

66EB7300-C73C-53C7-BFA5-AE266FBFD4F1

https://zoobank.org/B9F6FC6F-FA96-413D-9429-FD72CF8A1E23

**Figure 1.**
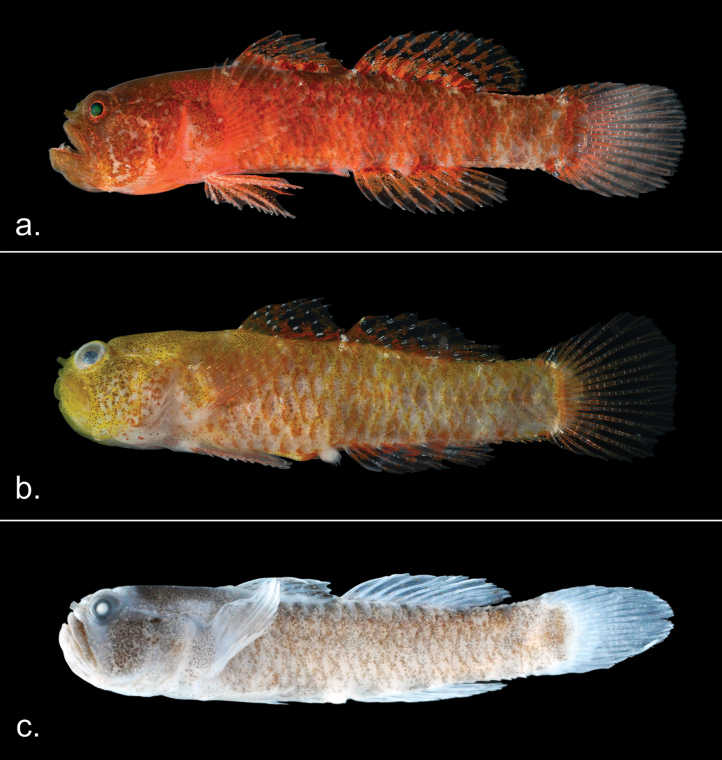
Specimens of *Sueviotaaethon* sp. nov. **a**UW 203365, holotype, freshly collected **b**UW 203367, freshly collected, showing the yellow variation of the species **c**UW 203365, holotype, preserved in 75% ethanol.

**Figure 2.**
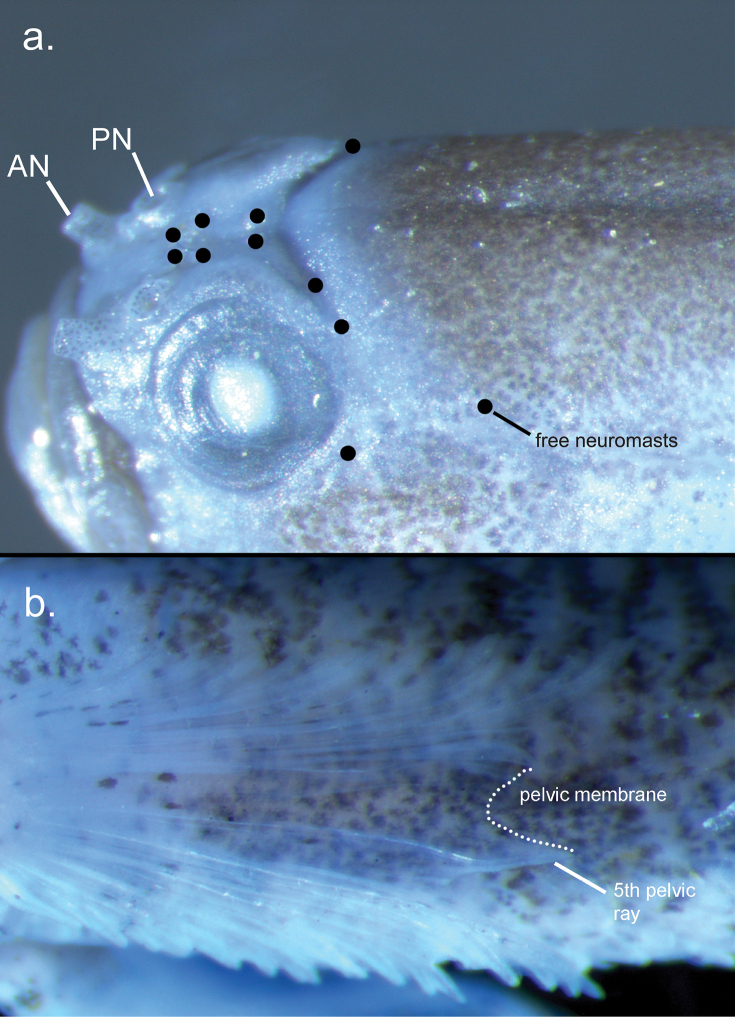
Morphological details of *Sueviotaaethon* sp. nov. holotype, UW 203365 **a** head details, showing the lack of cephalic sensory canals and location of free neuromasts (black dots). Anterior nares (AN) and posterior nares (PN) are labeled accordingly. **b** pelvic fin, showing the well-developed membrane and elongated fifth pelvic ray.

**Figure 3.**
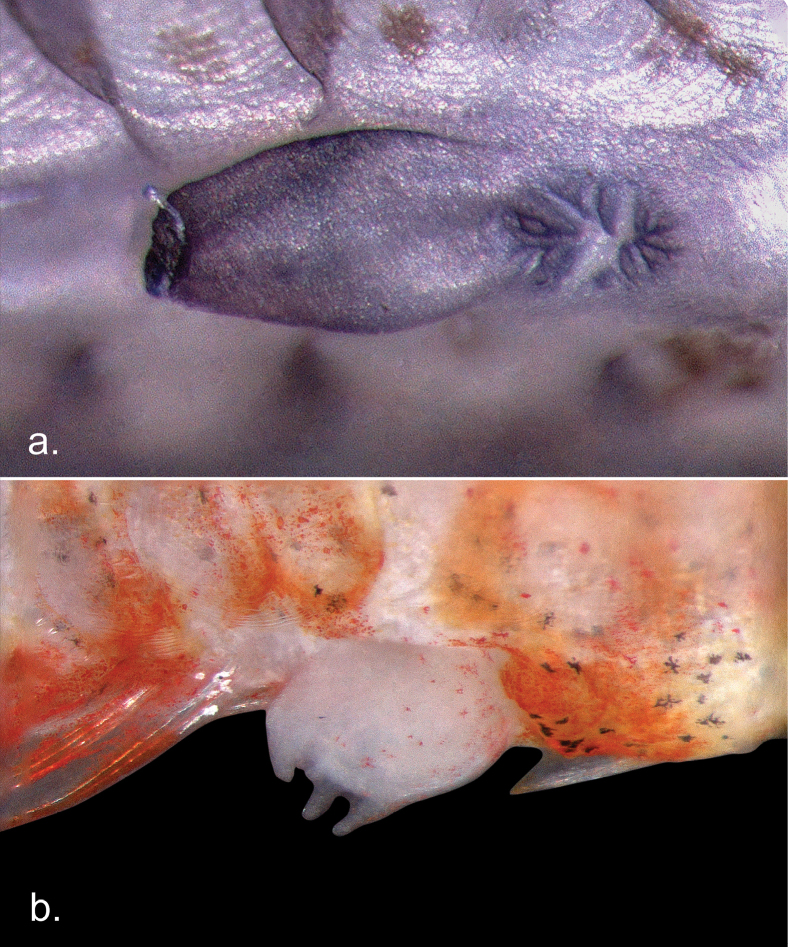
Urogenital papillae of *Sueviotaaethon* sp. nov. **a**UW 203366, male, preserved in ethanol and dyed with acid blue 113 **b**UW 203367, female, freshly collected. Right is anterior, top is dorsal.

**Figure 4.**
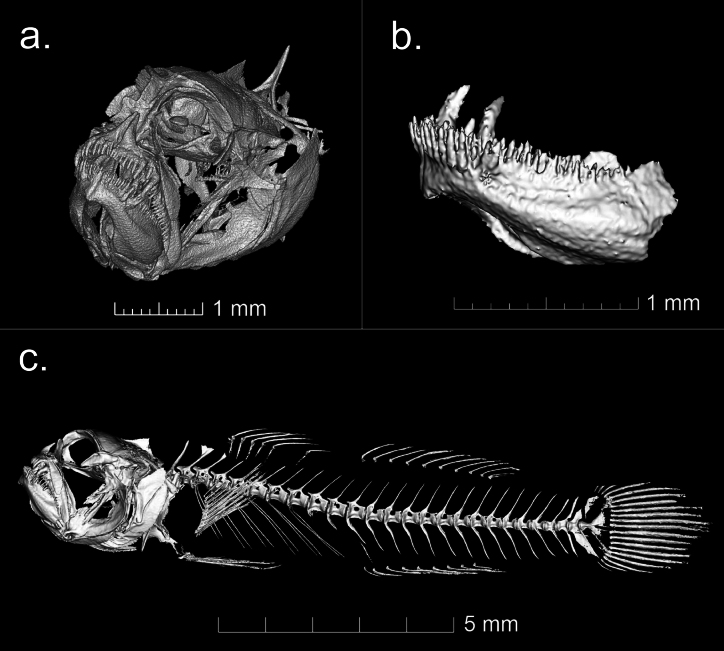
Micro-CT scan of *Sueviotaaethon* (UW 203365, holotype) showing its osteological characters **a** close-up of head showing the enlarged canines on the upper jaw **b** dentary, showcasing two enlarged canines in the internal row of teeth **c** lateral view of the complete skeleton.

Figs 1 , 2 , 3 , 4 Grumpy dwarfgoby

##### Type material.

***Holotype***. UW 203365, CRF186, female, 13.3 mm SL, Shark Reef 22.4283°N, 38.9932°E, central Red Sea, Thuwal, Makkah Province, Saudi Arabia, collected at 14 m from crustose coralline algae (CCA) covered overhang with lots of holes, clove oil and hand net, Viktor Nunes Peinemann, 22 June 2022. GenBank accession numbers: PP955452 (COI); PP955326 (16S). ***Paratypes***. • UW 203366, CRF114, male 13.3 mm SL, 19.8375°N, 39.9296°E, central Red Sea, Al Lith, Makkah Province, Saudi Arabia, collected at 30 m from the roof of a cave, clove oil and hand net, Viktor Nunes Peinemann, 19 May 2022. GenBank accession numbers: PP955451 (COI); PP955325 (16S). • UW 203367, CRF379, female, 12.4 mm SL, 22.2948°N, 39.0688°E, central Red Sea, Thuwal, Makkah Province, Saudi Arabia, collected at 10 m from a cave with a sandy bottom, collected as a mass sample, rotenone, Darren Coker, 7 August 2023. GenBank accession number: PP955453 (COI). • CAS-ICH 248441, CRF454, female, 9.2 mm SL, 18.8582°N, 40.3779°E, central Red Sea, Maras Reef, Makkah Province, Saudi Arabia, collected at 30 m from a CCA-covered overhang, clove oil and hand net, Viktor Nunes Peinemann, 6 June 2024. • CAS-ICH 248442, 6 specimens, 11.2 – 16.7 mm SL, 18.8582°N, 40.3779°E, central Red Sea, Maras Reef, Makkah Province, Saudi Arabia, collected at 20 m as a mass sample, rotenone, Viktor Nunes Peinemann, 6 June 2024.

##### Generic placement.

This species is placed in the genus *Sueviota* due to the presence of several key characteristics that differentiate *Sueviota* from the genus *Eviota*: a well-developed membrane between the fifth pelvic fin rays (extending entire length of ray) and the fifth pelvic rays elongate (77 – 88% of fourth ray), sometimes branched.

##### Diagnosis.

This is a species of *Sueviota* characterized by the following combination of characters: no cephalic sensory-canal pores; dorsal fin VI-I,8, or I,9, without filamentous spines; anal fin I,7 or I,8; pelvic fin I,5, rays 1 – 4 branched, fifth ray unbranched or with two branches, elongate (77–88% of fourth) and flattened towards the tips if unbranched, fourth ray longest; well-developed pelvic fin membrane fully joining fifth pelvic fin rays, frenum absent; 14 or 15 pectoral fin rays, some branched; body robust and deep, anterior slope of snout nearly vertical giving the head a blunt profile, terminal mouth inclined vertically forming a 72° angle to horizontal body axis.

##### Description.

Dorsal fin elements VI-I,9 (I,8), first dorsal fin rounded to square shaped, second and third spines slightly longer than the first spine; no elongate filaments on first dorsal fin; some or all soft rays of second dorsal fin branched, final ray branched to base; anal fin I,8 (one paratype I,7); pectoral fin rays 14 (14 – 15), 6 – 7 lower rays branched; pelvic fin I,5; fifth ray 77 – 88% of fourth ray; fourth pelvic fin ray with 4 branches, fifth pelvic fin ray unbranched or with 2 branches; 3-2-2 segments between consecutive branches of fourth pelvic fin ray; membrane connecting pelvic 5^th^ fin rays well developed extending out towards the tip, no frenum (Fig. 2B); 17 (15 – 17) segmented and 13 (12 – 13) branched caudal fin rays; lateral scale rows 25 (24 – 25); transverse scale rows 7 (6 – 7); ctenoid scales on body, no scales on head and breast; anterior extent of scales does not reach the base of the pectoral fin, no scales on the base of the first dorsal fin, but present on the base of the second dorsal fin; scales on the trunk extend ventrally onto the abdomen to beneath the pelvic fin rays with a small naked section on the ventral midline of the abdomen; front of the head distinctively blunt; mouth inclined vertically forming an angle of 72° to horizontal body axis, lower jaw projecting; upper jaw extending posteriorly to a vertical reaching the middle of the pupil; anterior tubular nares about 50% of the pupil diameter, posterior nares enlarged with an elevated rim adjacent to the eye; prominent canines; gill opening extending forward reaching the anterior edge of the operculum; lacking entire cephalic sensory canals and corresponding pores, but papillae (free neuromasts) are present where canal pores would be located (Fig. 2A); female urogenital papilla short and bulbous (Fig. 3B); male urogenital papilla elongated, smooth and bulbous with weakly fimbriate margin resembling type e (Fig. 3A); vertebral count 10 + 15 = 25 (Fig. 4C).

**Measurements** (percentage of SL; based on holotype and nine paratypes, 9.2 – 16.7): head length 27.1 (22.8 – 27.1); origin of first dorsal fin 33.8 (33.5 – 37.9), slightly behind pectoral fin base and pelvic fin origin; origin of second dorsal fin 55.6 (53.2 – 59.8); origin of anal fin 58.6 (56.2 – 60.9); caudal peduncle length 20.3 (19.5 – 23.8); caudal peduncle depth 15.0 (14.9 – 17.5); body depth at origin of first dorsal fin 22.6 (19.5 – 25.2), body relatively slender; pectoral fin length 16.5 (17.3 – 19.9); pelvic fin length 19.3 (15.0 – 21.3). As a percentage of HL: eye diameter 25.8 (22.2 – 30.8); snout length 15 (15.8 – 21.3); upper-jaw length 52.8 (46.9 – 59.3).

Teeth: *Sueviotaaethon* has two irregular rows of conical teeth in both its upper and lower jaws. As described by Winterbottom and Hoese (1988) in the initial description of the *Sueviota* genus, *S.aethon* has enlarged conical canines in both the upper and lower jaws. A closer examination of the head osteology through a micro-CT scan (Fig. 4) shows this detail with one enlarged curved canine situated on each side of the premaxilla, as well as one enlarged curved canine on each side of the innermost row of teeth on the dentary.

##### Fresh coloration.

The following description is based on ten specimens. Nine of these are dark red (Fig. 1A), while one is yellow-orange with several minor differences (Fig. 1B). Additional specimens are needed to determine the breadth of this intraspecific variation. Background of head and body bright red or yellow-orange; head and base of the pectoral fin heavily peppered with melanophores, giving the appearance of a burnt-red or peppered yellow coloration which intensifies towards nape and pre-dorsal area, melanophores concentrated on scale margins in yellowish specimen; cheeks heavily covered with melanophores with sparse marbling of white; no distinct spots on the pectoral fin base; breast, posterior margin of opercle, and branchiostegal areas light red in two specimens, white in a third; seven broad, faint, dark vertical bars on trunk starting from origin of first dorsal fin to base of caudal fin; two preanal bars poorly defined, five post-anal bars well defined and characterized by heavily pigmented subdermal melanophores; dark pigmentation of third and fourth bars extend to base of anal fin and give an appearance of two bright red spots on base of anal fin; no distinctive bars on head or radiating from the eye; fin elements on both dorsal and anal fins with alternating broad red and narrow white bands; pink band formed by tightly clustered chromatophores covers approximately one-third of dorsal and anal fins; dark red mottled pattern covering the remaining dorsal and anal fins; caudal fin primarily translucent except for relatively narrow pink bar of tightly clustered chromatophores on base of fin; pelvic fin translucent with small uniform light red chromatophores scattered along membrane but slightly condensed closer to the rays; pectoral fins translucent with no evident coloration; pupil of the eye black, and iris identical to the head coloration, with narrow iridescent gold ring surrounding pupil.

##### Color in preservative ethanol.

Background color of body and head pale whitish, covered by dark melanophores. Head, nape, and pre-dorsal areas heavily pigmented by tiny condensed melanophores extending along the dorsal region, melanophores more densely concentrated on scale margins; breast and branchiostegal areas brighter with fewer subdermal melanophores; seven dark bars along the trunk only barely distinguishable, the first six bars only slightly visible at dorsalmost and ventralmost edges, seventh bar located at base of caudal fin most apparent; fins generally translucent; membrane on external edge of first dorsal fin covered by tiny melanophores; some scattered melanophores on membranes of second dorsal fin and anal fin; no coloration on the pectoral, pelvic, and caudal fins (Fig. 1C)

##### Etymology.

The specific epithet stems from the ancient Greek Aethon, one of the four horses of the sun god Helios. The most similar species to *S.aethon*, *Sueviotapyrios* Greenfield & Randall, 2017, is named after a different horse of Helios. The specific name is a noun in apposition. The common name, Grumpy dwarfgoby, refers to the fish’s apparent grumpy and rather unhappy appearance, primarily due to the extremely upturned mouth position.

##### Distribution and habitat.

*Sueviotaaethon* is a rare species, with only ten specimens found during extensive rotenone and clove oil collections along the Saudi Arabian Red Sea coast. These specimens were collected at depths between 10 and 30 meters. A sample from another expedition (not presented here) was confirmed from 53 meters depth. The species may be more common at similar mesophotic depths, but further collections are needed to confirm this. The specimens collected with clove oil were found on CCA-covered roofs of small caves. Six specimens were collected from a single mass sampling at a CCA-covered wall with small crevices and holes. All but one of the specimens analyzed in this study were collected from exposed offshore reefs.

**Figure 5.**
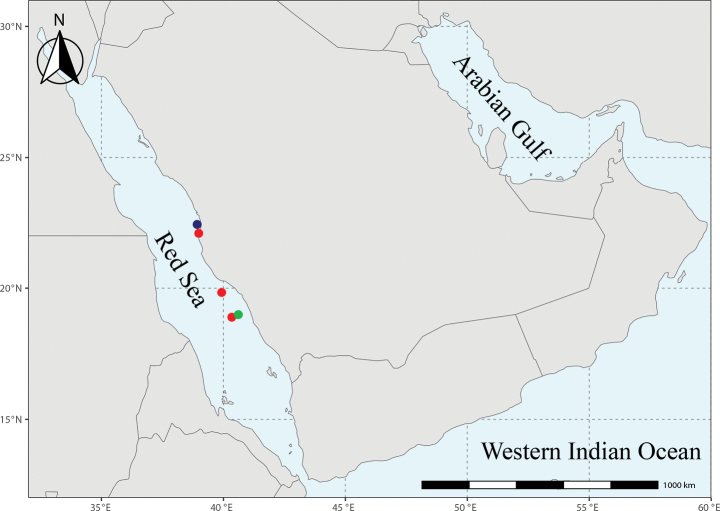
Map showing the localities where specimens were collected from. Blue: holotype, red: paratypes, green: confirmed non-type specimen.

*Sueviotaaethon* is presumably a Red Sea endemic. Our records range from Al Qunfudhah (18.9922°N, 40.6145°E) to Thuwal (22.4283°N, 38.9932°E), but it is likely that the species is more widely distributed throughout the main basin of the Red Sea (Fig. 5).

##### Comparisons.

A summary of key comparative characters in *Sueviota* spp. is provided in Table 1. *Sueviotaaethon* is distinguished from all other *Sueviota* spp. by its complete lack of cephalic sensory pores. The most similar species to *S.aethon* is *Sueviotapyrios*, which has a close and potentially overlapping geographic range, as well as a similar body shape, and coloration (Greenfield and Randall 2017). Besides the lack of cephalic sensory pores, *S.aethon* differs from *S.pyrios* by having no large red spots on dorsal and caudal fin elements, no elongate spines in the first dorsal fin, a shorter pelvic fin that does not reach the anus, branched pectoral fin rays, and a projecting lower jaw. *Sueviotapyrios* is currently known from only one specimen collected in the Gulf of Aqaba.

**Table 1.** Comparison of diagnostic morphological characters for species of *Sueviota*.

Species	Cephalic sensory pores	Elongate dorsal spines	Dorsal rays	Anal rays	Pectoral rays	Lateral scales	Pectoral fin	Pelvic rays 1–4	5^th^ pelvic ray	5^th^ pelvic ray length	Basal pelvic membrane	Pelvic frenum
*Sueviotaaprica*	NA, AITO, PITO, AOT	yes	10	8 to 9	17–19	26–27	branched	branched	branched	half of 4^th^	partial	no
*Sueviotaatrinasa*	NA, AITO, PITO, AOT, SOT	yes	9	7 to 8	17	24	branched	branched	branched	subequal to 4^th^	no	?
*Sueviotabryozophila*	POP, ITO*, SOT, AOT	no	8 to 9	7 to 8	16	24–25	unbranched	unbranched	branched	equal to 4^th^	yes	no
*Sueviotalachneri*	POP, NA, AITO, PITO, AOT, SOT	sometimes	9	8	16–18	25–26	branched	branched	branched	subequal to 4^th^	yes	no
*Sueviotalarsonae*	POP, NA, AITO, PITO, AOT, SOT	yes	9	7 to 8	17–19	21–24	branched	branched	branched	subequal to 4^th^	yes	yes
*Sueviotaminersorum*	POP, NA, AITO, PITO, AOT, SOT	yes	9	8	16–17 (17–18 in abstract)	27–28	branched**	branched	branched	subequal to 4^th^	yes	no
*Sueviotapyrios*	POP, NA, AITO, PITO, AOT, SOT	yes	8	8	16	25	unbranched	branched	branched	subequal to 4^th^	no	no
*Sueviotatubicola*	POP, NA, AITO, PITO, AOT, SOT	yes	9	8	19	26	branched	branched	branched	equal to 4^th^	yes	yes
*Sueviotaaethon* sp. nov.	none	no	8 to 9	7 to 8	14–16	24–25	branched	branched	unbranched or branched	77% to 88% of 4^th^	yes	no

* single interporbital pore, unclear as to whether it represents AITO or PITO; ** description says unbranched but photos of holoptype show branching.

Another superficially similar species is *Sueviotatubicola* Allen & Erdmann, 2017. *Sueviotaaethon* differs from *S.tubicola* by having no spots at the base of the pectoral fin, no cephalic sensory pores, no elongate dorsal spines, 14 – 15 pectoral rays (vs. 19 in *S.tubicola*), 5^th^ pelvic ray subequal in length to 4^th^ (vs. equal in *S.tubicola*), and no pelvic frenum (Allen and Erdmann 2017).

### ﻿Key to the species of *Sueviota*

**Table:** 

1	All cephalic sensory-canal pores absent (Red Sea) Grumpy dwarfgoby	***Sueviotaaethon* Nunes Peinemann, Pombo-Ayora & Tornabene, sp. nov.**
–	At least one cephalic pore present	**2**
2	(1) Cephalic sensory-canal pore system with POP, NA, AITO, PITO, SOT, and AOT pores	**3**
–	Cephalic sensory-canal pore system missing at least one of the pores above	**7**
3	(2) Pelvic frenum present	**4**
–	Pelvic frenum absent	**5**
4	(3) Two dark large spots on pectoral fin base, 26 longitudinal scales, 5^th^ pelvic fin ray length equal to 4^th^ (Papua New Guinea: Milne Bay) Tubeworm dwarfgoby	***Sueviotatubicola* Allen & Erdmann, 2017**
–	Spots on pectoral fin base absent, 21 – 24 longitudinal scales, 5^th^ pelvic fin ray length subequal to 4^th^ (northwestern Australia and South China Sea) Larson’s sueviota	***Sueviotalarsonae* Winterbottom & Hoese, 1988**
5	(3) Pectoral fin rays unbranched, lateral line scales 25 (Red Sea: Gulf of Aqaba) Fiery dwarfgoby	***Sueviotapyrios* Greenfield & Randall, 2017**
–	Pectoral fin rays branched, lateral line scales 21 – 28	**6**
6	(5) Caudal peduncle narrow, depth less than 60% caudal peduncle length, and less than 71% of body depth at anal-fin origin; lateral line scales between 25 – 26; 6 transverse scale rows (Indo-west Pacific) Lachner’s dwarfgoby	***Sueviotalachneri* Winterbottom & Hoese, 1988**
–	Caudal peduncle deep, depth greater than 60% caudal peduncle length, and greater than 75% of body depth at anal-fin origin; lateral line scales between 27 – 29, 7 transverse scale rows (Indonesia: West Papua) Miner’s dwarfgoby	***Sueviotaminersorum* Greenfield, Erdmann & Utama, 2019**
7	(2) Pelvic fin rays 1 to 4 unbranched, pectoral fin rays unbranched Bryozoan goby	***Sueviotabryozophila* Allen, Erdmann & Cahyani, 2016**
–	Pelvic fin rays 1 to 4 branched, pectoral fin rays branched	**8**
8	(7) SOT pores absent, 10 dorsal segmented rays, 26 – 27 lateral line scales, anterior nasal tube with black rim (Indo-west Pacific) Sunny dwarfgoby	***Sueviotaaprica* Winterbottom & Hoese, 1988**
–	SOT pores present, 9 dorsal segmented rays, 24 lateral line scales, anterior nasal tube entirely black (Western Australia) Blacknose dwarfgoby	***Sueviotaatrinasa* Winterbottom & Hoese, 1988**

## ﻿Discussion

The genera *Eviota* and *Sueviota* both contain very small fishes, even amongst gobies, with most adults being less than 20 – 30 mm SL. Species in these genera are superficially very similar to one another in terms of external morphology, general appearance, distribution, behavior and ecology. Most species in both genera lack scales on their head, nape, pectoral fin base, and have body coloration that generally consists of some pattern of red chromatophores and subcutaneous vertical bars (common) or horizontal stripes (less common) on the body. *Sueviota* was originally distinguished from *Eviota* by the following combination of characters: a basal pelvic membrane joining the fifth rays of the pelvic fins (vs pelvic fins well separate); the fifth pelvic rays themselves are very long (vs rudimentary or absent); and the fifth pelvic rays are branched (vs. unbranched) (Winterbottom and Hoese 1988). With the subsequent addition of five species to the genus since the original description, each of these characters now shows considerable variation, and there is not a single consistent synapomorphy for either genus that unambiguously differentiates the two. As such, it is unclear whether either genus is monophyletic (Winterbottom and Hoese 1988). The new species described here is the only *Sueviota* that completely lacks cephalic pores and canals, although generalized reduction in the development of cephalic canals is common throughout both *Eviota* and *Sueviota*, and several species of *Eviota* lack canals and pores entirely as well. Tornabene et al. (2013) demonstrated that pore patterns are highly variable and homoplasious across *Eviota* – a trend that likely applies to *Sueviota* as well. We are currently evaluating the phylogenetic relationships among these two genera using molecular data, but refrain from making conclusions at this point until a more comprehensive sampling of both genera, as well as potentially closely related genera, can be achieved. It is highly likely that the genetic classification for at least some *Eviota* and *Sueviota* species, including possibly this species, may change as we have a clearer understanding of the phylogeny of this group.

## Supplementary Material

XML Treatment for Sueviota aethon

## ﻿Additional information

### Author ORCIDs

Viktor Nunes Peinemann https://orcid.org/0000-0002-7060-397X

Lucía Pombo-Ayora https://orcid.org/0000-0003-0316-0280

Luke Tornabene https://orcid.org/0000-0002-0673-2320

Michael L. Berumen https://orcid.org/0000-0003-2463-2742

### Data availability

All of the data that support the findings of this study are available in the main text.
